# Nesfatin-1 regulates the phenotype transition of cavernous smooth muscle cells by activating PI3K/AKT/mTOR signaling pathway to improve diabetic erectile dysfunction

**DOI:** 10.1016/j.heliyon.2024.e32524

**Published:** 2024-06-16

**Authors:** Keming Chen, Bincheng Huang, Jiajing Feng, Shuzhe Fan, Zhengxing Hu, Shuai Ren, Haifu Tian, A.L.-QAISIMOHAMMED Abdulkarem, Xuehao Wang, Yunshang Tuo, Xiaoxia Liang, Haibo Xie, Rui He, Guangyong Li

**Affiliations:** a Urology Department of General Hospital, Ningxia Medical University, Ningxia 750000, China; b Key Laboratory of Fertility Preservation and Maintenance of Ministry of Education, Ningxia Medical University, Yinchuan, China; c School of traditional chinese medicine NingxiaMedicalUniversity,Yinchuan, China

**Keywords:** Nesfatin-1, Corpus cavernosum smooth muscle cells, Type 2 diabetes, Erectile dysfunction, Metabolic disorder

## Abstract

**Objective:**

This study aims to explore the impact of Nesfatin-1 on type 2 diabetic erectile dysfunction (T2DMED) and its underlying mechanism in regulating the phenotypic switching of corpus cavernosum smooth muscle cells (CCSMCs).

**Methods:**

Twenty-four 4-week-old male C57 wild-type mice were randomly assigned to the control group, model group, and Nesfatin-1 treatment group. Monitoring included body weight, blood glucose levels, and penile cavernous pressure (ICP). Histochemistry and Western blot analyses were conducted to assess the expressions of α-SMA, OPN, and factors related to the PI3K/AKT/mTOR signaling pathway. CCSMCs were categorized into the control group, high glucose and high oleic acid group (GO group), Nesfatin-1 treatment group (GO + N group), sildenafil positive control group (GO + S group), and PI3K inhibitor group (GO + N + E group). Changes in phenotypic markers, cell morphology, and the PI3K/AKT/mTOR signaling pathway were observed in each group.

**Results:**

(1) Nesfatin-1 significantly ameliorated the body size, body weight, blood glucose, glucose tolerance, and insulin resistance in T2DMED mice. (2) Following Nesfatin-1 treatment, the ICP/MSBP ratio and the peak of the ICP curve demonstrated a significant increase. (3) Nesfatin-1 significantly enhanced smooth muscle and reduced collagen fibers in the corpus cavernosum. (4) Nesfatin-1 notably increased α-SMA expression and decreased OPN expression in CCSMCs. (5) Nesfatin-1 elevated PI3K, *p*-AKT/AKT, and *p*-mTOR/mTOR levels in penile cavernous tissue.

**Conclusions:**

Nesfatin-1 not only effectively improves body weight and blood glucose levels in diabetic mice but also enhances erectile function and regulates the phenotypic switching of corpus cavernosum smooth muscle. The potential mechanism involves Nesfatin-1 activating the PI3K/AKT/mTOR signaling pathway to induce the conversion of CCSMCs to a contractile phenotype.

## 1 Introduction

With the improvement of living standards, the incidence of type 2 diabetes is gradually increasing. It is estimated that by 2045, there will be around 700 million people living with diabetes globally [1]. In a representative sample of Chinese adults, the prevalence of diabetes was estimated to be 11.6 %, and the prevalence of prediabetes was 50.1 % [2]. These evidences indicate that diabetes mellitus has become an important public health problem. The complications of diabetes mellitus (DM) have always been a serious health problem. Long-term complications of type 2 diabetes include microangiopathy, neuropathy, and sexual dysfunction (SD). Erectile dysfunction (ED) has long been regarded as the most important male sexual dysfunction. A cross-sectional study of 372 men showed a significant correlation between diabetes and erectile dysfunction, and it was extremely common in the diabetic population [3]. In recent years, with the improvement of living standards, the incidence of type 2 diabetes mellitus erectile dysfunction has increased, which seriously affecting people's quality of life [2]. At the same time, the current treatment of T2DMED mainly relies on strict diet and effective drug control of blood glucose, correction of dyslipidemia and control of hypertension, at the same time oral phosphodiesterase type 5 inhibitors and penile cavernous drug injection therapy are required to achieve a certain effect.Currently, there is a lack of drugs that can simultaneously control blood glucose and improve erectile dysfunction [4]. The precise mechanism underlying erectile dysfunction resulting from type 2 diabetes remains unclear. This lack of clarity is also responsible for the limited efficacy observed when simply administering drugs to manage blood glucose and address erectile dysfunction in clinical settings.

The American Association of Urological Surgeons guidelines define erectile dysfunction as a persistent or recurring condition in which the penis is unable to erect and/or maintain an erection to complete satisfactory sexual intercourse [5]. It is one of the most common multifactorial diseases of the male reproductive system in male sexual dysfunction. Penile erection mainly depends on dilating the cavernous artery and cavernous sinus.The contraction and relaxation of CCSMs determine the expansion of the cavernous sinus and penile erection. Therefore, maintaining the normal function of corpus cavernosal smooth muscle plays a crucial role in improving penile erection and the treatment of T2DMED. Studies have shown that the smooth muscle phenotype is an important factor in maintaining the function and structure of smooth muscle, and regulating the phenotype of corpus cavernosum smooth muscle to the contractile type can improve the occurrence of ED [6]. At present, a large number of literature confirmed hypoxia [[7], [8], [9]], platelet-derived growth factor [10], chronic prostatitis [11], tobacco harmful gas [12], and other external factors may lead to the transition of CCSMs phenotype to synthetic phenotype, and then lead to ED. Diabetes hyperglycemia is also an important factor leading to smooth muscle phenotype switching [13], however, it is rarely reported in T2DMED.

Nesfatin-1 is a newly discovered secretory peptide found in the hypothalamus, brain stem, and gastrointestinal tract. It is a hydrolytic fragment of the nuclear group of protein 2 (NUCB2). In recent years, as research on diabetes-related complications has advanced, the role of Nesfatin-1 as a novel peptide molecule has gained widespread recognition. It has been acknowledged for its ability to enhance glucose metabolism, reduce blood glucose levels, regulate cardiovascular function, and consequently, ameliorate diabetes complications along with associated structural and functional abnormalities in organs [14]. Current studies have confirmed that Nesfatin-1 can improve diabetic cardiomyopathy [15], diabetic nephropathy [16], diabetic retinopathy [17], diabetic polycystic ovary syndrome [18,19] and other diseases, which is considered as one of the important potential target drugs for the treatment of diabetes-related complications [20](Prinz and Stengel, 2016).However, there have been no reports on whether Nesfatin-1 can improve Type 2 Diabetes Mellitus Erectile Dysfunction (T2DMED) and its specific mechanism. Therefore, this study aims to investigate the potential of Nesfatin-1 to improve T2DMED and elucidate its mechanism by examining the regulation of Nesfatin-1 on the phenotype switching of cavernous smooth muscle cells. The objective is to offer new insights for the clinical treatment of T2DMED.

## 2 Materials and methods

### 2.1 Experimental materials and instruments

#### 2.1.1 Experimental animals and cell culture

Twenty-four SPF grade 4-week-old wild-type C57BL/6J male mice weighing about 20g±3g were purchased from the Laboratory Animal Center of Ningxia Medical University. (Certificate number: SCXK2020-0001). Feeding conditions: constant temperature and humidity, 12 h of light alternating daily, free feeding and drinking, quiet environment. Corpus cavernosum smooth muscle cells (CCSMCs) (Item No. CP-M218), Purchased from Wuhan Punosai Company. The first 5 generations of cultured cells were used in the experiment.

#### 2.1.2 1.2.Experimental reagents and instruments

High-fat Diets were obtained from Research Diets, USA (60 Kcal% Fat, Lot number:D12492; Nesfatin-1 (Item No.H-003-22) purchased from Phoenix (Burlingame, CA, USA); Immunohistochemical reagents were purchased from Beijing Zhongshan Jinqiao Biotechnology Development Co.LTD.OPN, α-SMA, PI3K, AKT, *p*-AKT, mTOR, *p*-mTOR antibodies and goat anti-rabbit and mouse antibodies secondary antibodies were purchased from Affinity Biosciences. Oleic acid (Item No. HY-N1446), Esculetin (Item No. HY-NO284); Sildenafil (Item No. HY-15025, 10 mM), all bought in MCE company.

## 3 Experimental methods

### 3.1 Animal experiments

#### 3.1.1 Animal models were established and grouped

Twenty-four 4-week-old wild-type C57BL/6J male mice weighing about 20g±3g were randomly divided into Control group, Model group, and Nesfatin-1 treatment group, with 8 mice in each group.The control group was fed with a normal diet, and the model group and Nesfatin-1 treatment group were fed with a 60 % high-fat diet for 16weeks. After 16weeks, Nesfatin-1 treatment group was intraperitoneally injected with Nesfatin-1 (8 nM/kg) for 4 weeks, and model group was fed with high-fat diet for 4 weeks.All animal experiments were approved by the Ethics Committee of Ningxia Medical University.Nesfatin-1 preparation: the powder of Nesfatin-1 (1mg/fork) was diluted by adding 1 ml normal saline, and the working solution was prepared by shaking and mixing. The mice were weighed in advance and the dosage required for each injection was calculated according to the concentration of 8 nM/kg. According to the calculated total dose required for each injection, the appropriate working solution was drawn and diluted again with normal saline to the required total dose. The amount of injection required for each mouse was calculated and intraperitoneally injected.

#### 3.1.2 Body mass, blood glucose monitoring, and intracavernous pressure (ICP) measurement were performed

Body mass:The body mass of mice was monitored every 7 days during the same time period to analyze body mass changes.blood glucose:Oral glucose tolerance test OGTT: After fasting for 16–18h before sampling, the mice were given 3 g/kg glucose solution by gavage, and blood glucose was measured at 0min, 15mins, 30mins, 60mins, 90mins, and 120mins. The changes of blood glucose and the area under the curve AUC were recorded and analyzed.Insulin tolerance test ITT: After fasting for 5 h for 2 days before sampling, the body weight was measured, and the blood glucose was measured at 0min, 15mins, 30mins, 60mins, and 120mins after intraperitoneal injection of insulin (1 IU/kg), and the AUC was analyzed.

#### 3.1.3 Intracavernous pressure (ICP)

After the mice were anesthetized with 2 % pentobarbital sodium 45 mg/kg and routinely sterilized. The foreskin and tunica albuginea were separated, and the subcutaneous fat and connective tissue were free until the penile cavernous nerve was obviously exposed. A platinum bipolar electrode was connected to an electrical stimulator, and the cavernous nerve was stimulated at 5V for 1min. The changes of intracavernous pressure during electrical stimulation were observed and recorded. The mean systemic blood pressure (MSBP) and the maximum ICP during the stimulation of the erectile nerve were measured and the ratio of ICP to MSBP was compared to evaluate the erectile response.

#### 3.1.4 The level of glycosylated hemoglobin (HbA1c) in serum was detected by ELISA

Serum samples of mice in each group were collected, and anticoagulant was added and centrifuged. The levels of glycosylated hemoglobin (HbA1c) in serum samples of mice in each group were detected by ELISA method, and the operation steps were strictly according to the instructions in the kit.

#### 3.1.5 Immunohistochemical staining

After dewaxing and entry, the paraffin sections were rinsed 3 times with PBS, and then the antigen was repaired with 0.01 mol/L citrate buffer for 15 min. After natural cooling for 2h, the sections were rinsed again, and the surface was covered with endogenous peroxidase blocker for 30mims. After rinsing, the sections were blocked with goat serum at room temperature for 1h, and the primary antibody was dropped and then blocked at 4 °C overnight. The next day, a secondary antibody was added after rewarming for 30 min, incubated at room temperature for 1h, and DAB was used for color development. After color development was completed, the slides were stained with hematoxylin solution for 4 min, differentiated with 1 % hydrochloric acid alcohol, flushed for 15 min, and sealed with neutral resin after routine dehydration.

#### 3.1.6 Animal tissues were detected by Western Blotting

After a freezing mill fully ground the penile tissue, the supernatant was taken, and the protein was extracted from the penile tissue of mice by the NDA/RNA/Protein Kit (Item No. R6734–01, omega Company). The protein concentration was measured according to the instructions of the BCA protein detection kit, and the loading volume was calculated. Electrophoresis at 80V voltage was followed by a 200 mA electric transfer of 150mims. After blocking with 5 % skim milk for 2h, the primary antibody was incubated at 4 °C overnight, and the next day, the secondary antibody was incubated at room temperature for 2h. Images were acquired after exposure and quantified for analysis.

### 3.2 Cell experiments

#### 3.2.1 Culture and processing of mouse corpus cavernosum smooth muscle cells

CCSMCs were routinely cultured in DMEM/F12 substrate containing 10%FBS.

#### 3.2.2 Cell proliferation was determined by CCK-8 assay to select each drug's treatment time and concentration

CCSMCs were routinely cultured and treated with Nesfatin-1 at concentrations of 0 ng/ml, 50 ng/ml, 100 ng/ml, 150 ng/ml, 200 ng/ml, 250 ng/ml and 300 ng/ml. Oleic acid at Concentration of 0 nM, 2.5 nM, 5 nM, 10 nM, 15 nM, 20 nM. The concentration of Esculetin was 0 μM, 10 μM, 20 μM, 30 μM, 40 μM; Each drug and concentration were set up 0h, 12h, 24h, 48h, 72h time gradient. Combined with the previous experiments, the concentration and time with the greatest change in cell OD were selected for further experiments.

#### 3.2.3 Cellular immunofluorescence staining experiments

CCSMCs were cultured on cover slides. When the density reached 90 %, it was fixed in 4 % paraformaldehyde for 30mins, translatated with 0.3 % Triton X-100 for 20mins, sealed with 5 % BSA for 1h, the primary antibody was incubated at 4 °C overnight, and the secondary antibody was incubated with fluorescein labeled at room temperature for 2h, and then sealed with DAPI-containing tablets.

#### 3.2.4 Western blot was used to detect the expression of corresponding proteins in CCSMCs

The grouped treated cells were fully lysed with RIPA lysate for 30mims, adherent cells were treated with a cell scraper, centrifuged, and the supernatant was removed for determination of protein concentration by BCA and fixed with Buffer. Electrophoresis was performed at 80V with a 200 mA electrotransfer and blocked with skim milk. The primary antibody was incubated at 4 °C overnight, and the secondary antibody was incubated for 2h. After exposure, images were acquired and quantified for analysis.

## 4 Statistical methods

Graph Pad Prism 9.0 software was used to analyze the experimental results. Data were expressed as mean ± standard deviation (mean ± SD). One-way variance analysis was used for comparison of means between groups, and the Dunnett test was used for pairwise comparison of homogeneity of variance. P < 0.05 was considered statistically significant.

## 5 Result

### 5.1 1.The type 2 diabetes mellitus erectile dysfunction (T2DMED) mouse model was induced through a 16-week high-fat feeding protocol. The exogenous administration of Nesfatin-1 demonstrated an improvement in the body weight and glucose metabolism indices of the T2DMED mice

**Fig. 1:**
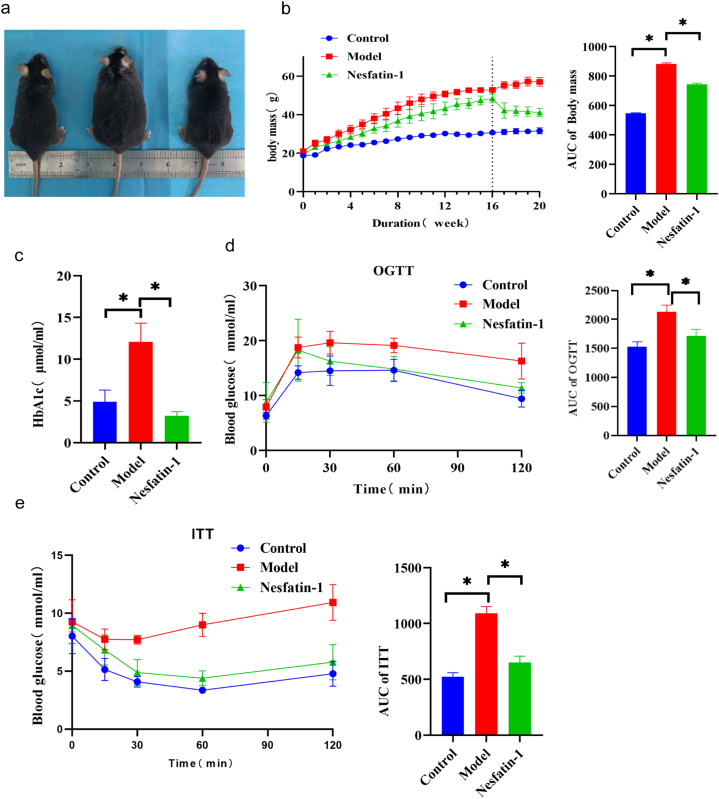
Changes in metabolism-related indicators in mice. a.Changes in the mouse body size; b.Changes in mice body mass and AUC in each group of mic; c.Changes of fasting blood glocuse in each group of mice; d.Changes of HbA1c in each group of mice; e.Changes in OGTT value and AUC in each group of mice; f.Changes in ITT value and AUC in each group of mice. (OGTT:Oral glucose tolerance test; ITT:Insulin tolerance test; AUC:area under the curve; n = 5 per group, *P＜0.05).

After 16 weeks of high-fat feeding, the mice became fatter, weight increased significantly, and fasting blood glucose increased.The results of oral glucose tolerance test (OGTT) and insulin tolerance test (ITT) showed that blood glucose was significantly higher than that of normal control group at 15, 30, 60 and 120 min. Exogenous give Nesfatin-1 4 weeks after treatment, the body weight of the mice was significantly lower than that of the model group (P = 0.0001), and the blood glucose, impaired glucose tolerance and insulin resistance of the mice were significantly improved (Fig. 1a-c) (Body mass: Control group: pre-treatment 52.88 ± 1.1g,post-treatment 31.56 ± 1.6g; Model group: pre-treatment 48.4 ± 2.2g,post-treatment 57.00 ± 2.2g; Nesfatin-1 group: pre-treatment 49.7 ± 1.2g,post-treatment 41.1 ± 2.2g. Fasting blood glucose: Control group:5.0 ± 0.5; Model group:9.4 ± 1.5; Nesfatin-1 group:5.7 ± 0.2). Glycated hemoglobin (HbA1c) levels compared with model group significantly reduced (Control group: 4.9μ±1.4 mol/ml; Model group: 11.1μ±3.1 mol/ml; Nesfatin-1 group: 3.2μ±0.5 mol/ml). OGTT and TII results of mice treated with Nesfatin-1 were significantly improved.The oral glucose tolerance test (OGTT) and insulin tolerance test (ITT) showed that blood glucose levels were significantly higher at 15, 30, 60, and 120 min compared to the control group. After 4 weeks of Nesfatin-1 treatment, the OGTT and ITT results in the treatment group significantly decreased (Fig. 1d-e), with statistically significant differences (P < 0.05). Additionally, the level of glycated hemoglobin (HbA1c) after Nesfatin-1 treatment was significantly lower than the model group (P < 0.05) (Fig. 1c) (Control group: 4.9μ±1.4 mol/ml; Model group: 11.1μ±3.1 mol/ml; Nesfatin-1 group: 3.2μ±0.5 mol/ml). with statistically significant differences (P < 0.05).

### 5.2 2.T2DMED mice, subjected to a high-fat diet for 16 weeks, exhibited the development of erectile dysfunction. Notably, the exogenous administration of Nesfatin-1 significantly ameliorated erectile dysfunction in T2DMED mice

**Fig. 2:**
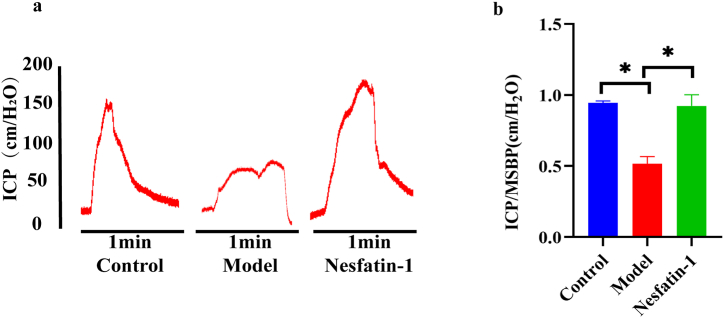
The changes of erectile function in mice. a.ICP curves in mice. b.Changes in the ratio of ICP/MSBP of mice in each group. (ICP:Intracaversal pressure; MSBP:Systemic blood pressure (MSBP); n = 4 per group *P＜0.05).

The peak value of the ICP curve in normal mice was 155 ± 13.1cmH_2_O, and the mean value of ICP/MSBP was 0.90 ± 0.05, while the peak value of ICP curve in T2DMED mice was significantly reduced to 80 ± 22cmH_2_O, and the mean value of ICP/MSBP was 0.49 ± 0.13. The difference was statistically significant (P < 0.05). After Nesfatin-1 treatment, the peak value of ICP curve in mice increased significantly and the ICP curve became steeper (Fig. 2a). The average peak value of the curve was 154.4 ± 16.1cmH_2_O, and As the average of ICP/MSBP was 0.9 ± 0.04. Further statistical comparison of ICP/MSBP showed statistical differences among the three group (Fig. 2b) (P < 0.05).

### 5.3 3.Nesfatin-1 increases the smooth muscle content of T2DMED mice

**Fig. 3:**
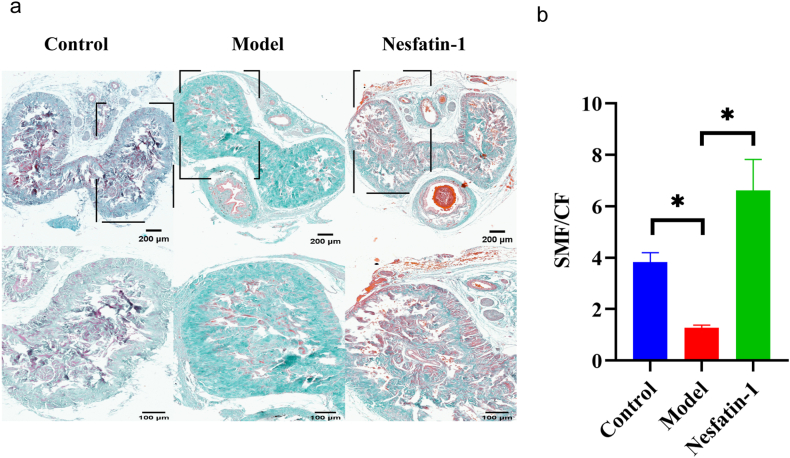
The content of smooth muscle fibers and collagen in mice. a.Masson staining results in each group of mice. (up bar: 200 μm; down bar:100 μm); b.Changes in the ratio of smooth muscle fiber to collagen fiber content in mice. (n = 4 per group *P＜0.05).

Masson staining was used to observe the changes of in smooth muscle and collagen fibers in the corpus cavernosum. We found that the smooth muscle fibers in T2DMED mice were significantly less than those in the control group, and the content of collagen fibers was significantly increased. After 4 weeks of Nesfatin-1 treatment, the smooth muscle increased and the collagen fibers decreased (Fig. 3a-b), with statistically significant differences (P < 0.05).

### 5.4 4.Nesfatin-1 regulates the corpus cavernosal smooth muscle phenotype in T2DMED mice

**Fig. 4:**
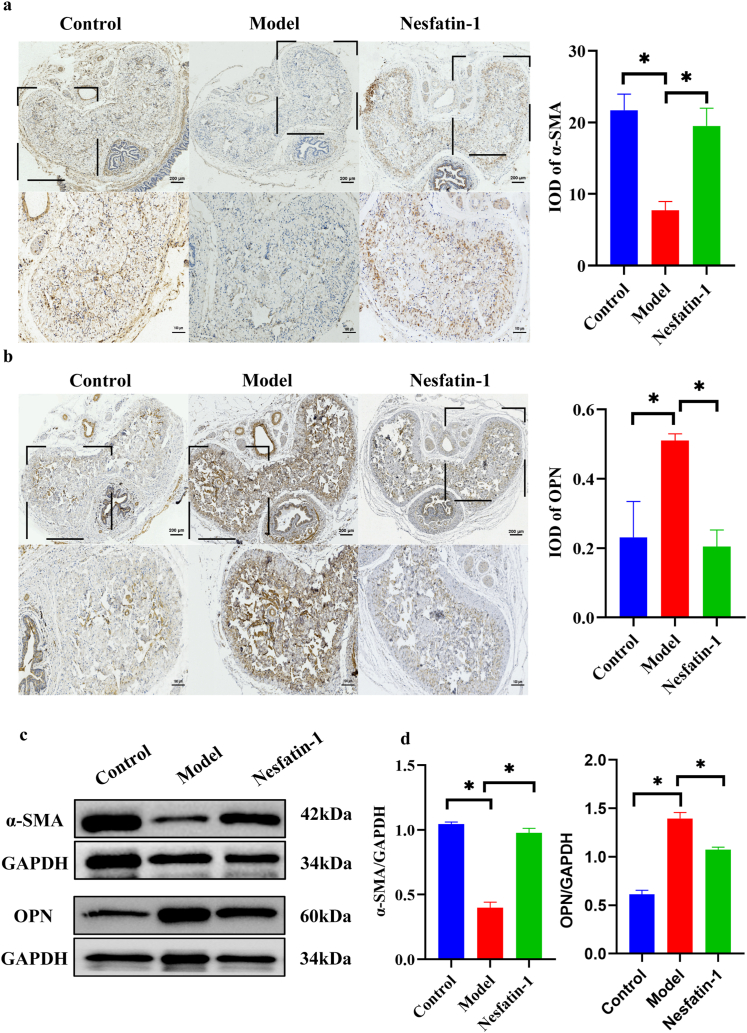
The expression of phenotypic markers in mice. a.Immunohistochemical micrographs and quantitative analyses of α-SMA in mice; b.Immunohistochemical micrographs and quantitative analyses of OPN in mice; (up bar: 200 μm; down bar: 100 μm); c.The level ofα-SMA and OPN in penile cavernous tissue of mice was determined by Western blot; d.Quantitative analysis of α-SMA and OPN by Western blot. (n = 4 per group *P＜0.05).

Immunohistochemistry showed that α-SMA, a marker of smooth muscle contraction phenotype, significantly decreased in T2DMED mice and increased significantly after Nesfatin-1 treatment. However, the synthetic phenotypic marker OPN was significantly increased in the model group and decreased significantly by Nesfatin-1 treatment (Fig. 4a-b). Further quantitative analysis of α-SMA and OPN by Western blot showed that the change trend was consistent with the results of immunohistochemistry (Fig. 4c-d).

### 5.5 5.Nesfatin-1 regulates the PI3K/AKT/mTOR signaling pathway

**Fig. 5:**
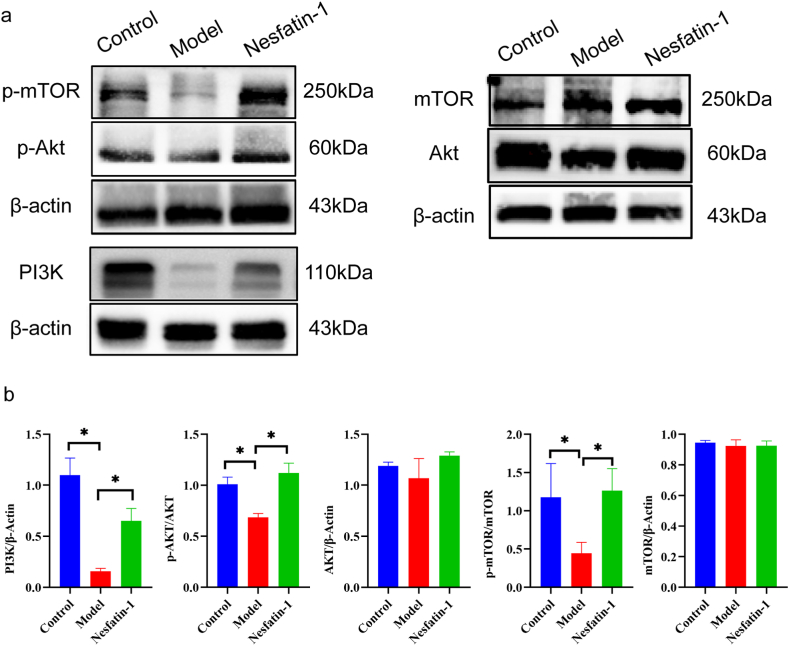
Effect of PI3K/AKT/mTOR signaling pathway on phenotypic switching of corpus cavernosum smooth muscle. a.The level of PI3K,p-AKT,AKT, *p*-mTOR and mTOR in the penile cavernous tissue of mice was determined by Western blot. b.Quantitative analysis of PI3K,p-AKT,AKT, *p*-mTOR and mTOR in each group (n = 4 per group *P＜0.05).

The protein level PI3K, *p*-AKT/AKT and *p*-mTOR/mTOR in the penile cavernous tissue of mice was remarkably decreased in T2DMED (P < 0.05). Compared to the model group, the level of PI3K, *p*-AKT/AKT and *p*-mTOR/mTOR in penile cavernous tissue was remarkably increased in the treatment group (P < 0.05). Western blot results and quantitative analysis of gray values of bands showed that the PI3K was inhibited in T2DMED mice, and the phosphorylation levels of its downstream factors AKT and mTOR were also significantly decreased, but the total AKT and mTOR did not change. After Nesfatin-1 treatment, PI3K was significantly activated in the treatment group, and its downstream *p*-Akt and *p*-mtor were also significantly increased (P < 0.05), but there was no significant difference in the total AKT and mTOR levels (Fig. 5a-b).

### 5.6 6.The cell model was established through co-culture with high glucose and high oleic acid. Sildenafil, Nesfatin-1, and Esculetin were administered to observe their effects on the morphology and quantity of CCSMCs

**Fig. 6:**
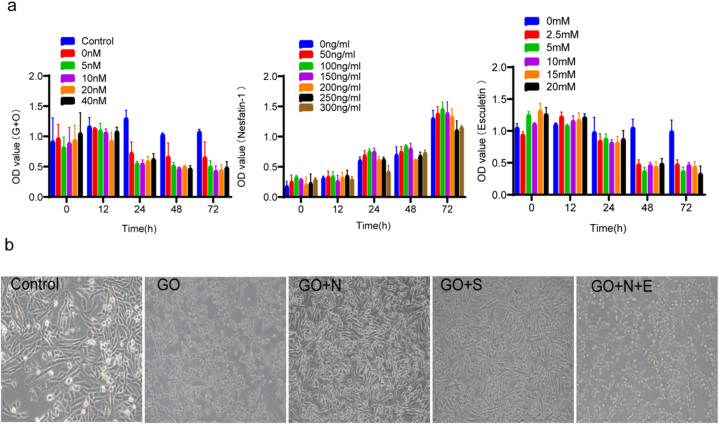
CCK-8 results of oleic acid, nesfatin-1 and Esculetin and micrographs of corpus cavernosum smooth muscle cells in each group after treatment. a.DO values of oleic acid, nesfatin-1, and Esculetin in cck-8 assay. b.Micrographs of spongy smooth muscle cells in each group after 48 h of treatment. (magnification:400×).

CCSMCs were detected by CCK-8 experiment according to the corresponding drug concentration gradient and time gradient, and the corresponding optical density (OD) value of each hole measured at 450 nm was determined. The results showed that the concentration of Acid oil was 20 mM; The concentration of Nesfatin-1 at 150 ng/mL; Esculetin at 40 μM and treated separately for 48 h had the greatest effect on OD values (Fig. 6a). Therefore, we selected the drug concentration and treatment time for subsequent experiments.

CCSMCs were divided into groups according to the drug concentration and treatment time selected by CCK-8 assay, and pictures of each group were taken by inverted microscope camera system. CCSMCs treated with high glucose and high oleic acid were round in shape and decreased in number. After Nesfatin-1 and Sildenafi treatment, most of the cells returned to spindle shape and their number increased significantly. There was no significant difference in cell morphology and number between Nesfatin-1 and Sildenafi treatment groups. However, after treatment with PI3K/AKT inhibitor Esculetin, the morphology of CCSMCs returned to the main round shape and the number of CCSMCS decreased significantly (Fig. 6b).

### 5.7 7.Through in vitro cell experiments, it has been confirmed that Nesfatin-1 regulates the transformation of CCSMCs into a contractile type, with the PI3K/AKT/mTOR signaling pathway playing a crucial role in this process

**Fig. 7:**
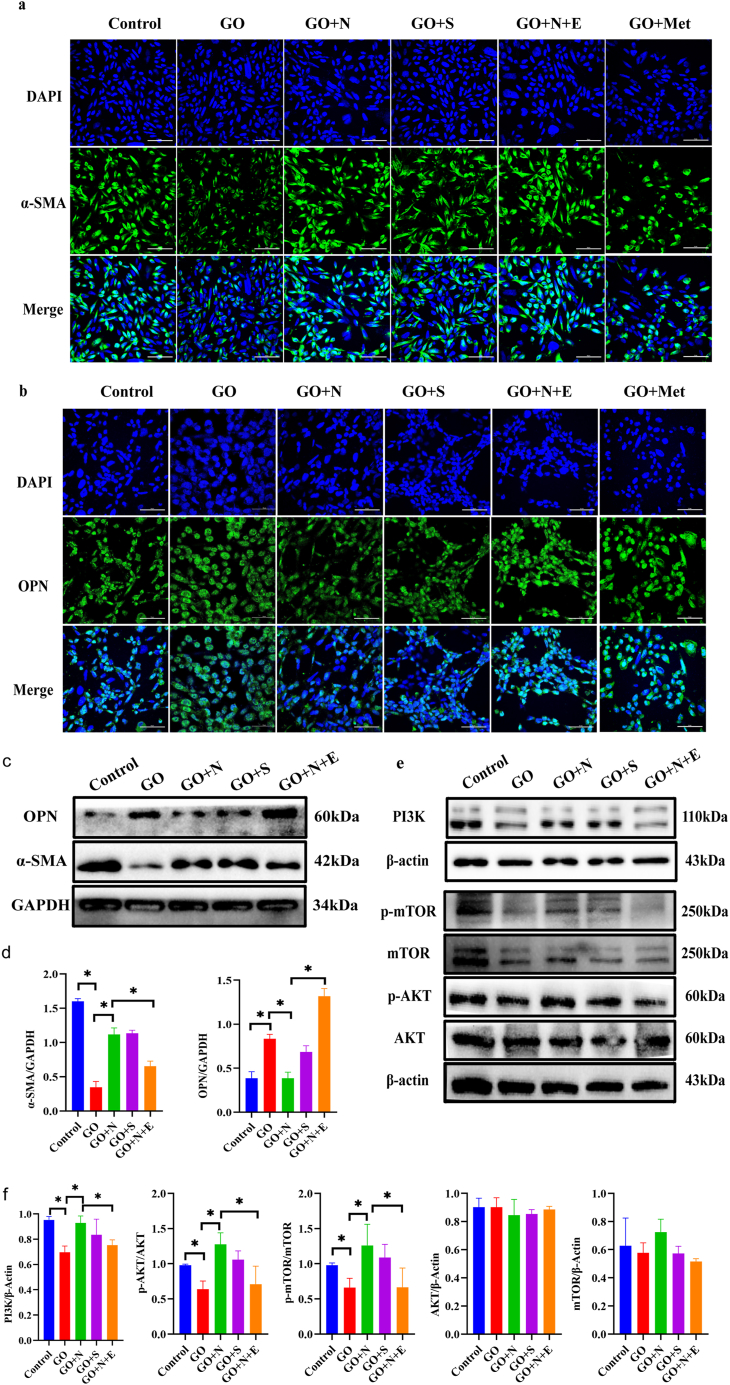
The expression changes of CCSMCs phenotypic markers and PI3K/AKT/mTOR signaling pathway. a.α-SMA expression in CCSMCs by immunofluorescence (Bar 50 μm); b:OPN expression in CCSMCs by immunofluorescence. (Bar 50 μm); c.The level ofα-SMA and OPN in penile cavernous tissue of mice were determined by Western blot; d.Quantitative analysis of α-SMA, OPN in each group; e.The level of PI3K,p-AKT,AKT, *p*-mTOR and mTOR in the penile cavernous tissue of mice was determined by Western blot; f.Quantitative analysis of PI3K,p-AKT,AKT, *p*-mTOR and mTOR in each group. (n = 4 per group *P＜0.05).

Immunofluorescence indicated that α-SMA expression decreased significantly in GO group, while OPN expression increased significantly and its shape changed from spindle to round. After Nesfatin-1 and Sildenafi treatment, α-SMA increased significantly, OPN decreased significantly, and cell morphology returned to a spindle shape. However, α-SMA expression was not significantly increased and OPN expression was not significantly decreased in the GO + N + E group, showing a trend similar to that in the GO group (Fig. 7a-b). We quantitatively analyzed α-SMA and OPN of cells in each group by Western blot, and the change trend was consistent with the immunofluorescence results (Fig. 7c-d). We quantitatively analyzed the PI3K/AKT/mTOR signaling pathway expression of CCSMCs in each group by Western blot. The results indicated that the expressions of *p*-AKT/AKT and *p*-mTOR/mTOR were significantly increased in CCSMCs treated after Nesfatin-1 and Sildenafi treatment (P < 0.05). However, the expressions of *p*-AKT/AKT and *p*-mTOR/mTOR in the GO + N + E group were significantly decreased compared with those in GO + N and GO + S groups (P < 0.05), showing a trend similar to that in the GO group (Fig. 7e-f).

## 6 Discussion

Currently, more than half of diabetes patients worldwide are suffering from ED, and diabetes is gradually recognized as an important cause of ED [21,22]. The clinical treatment of diabetes-related erectile dysfunction mainly relies on basic therapies such as dietary control, hypoglycemic medication, combined with oral phosphodiesterase type 5 inhibitors (tadalafil/sildenafil, etc.) and penile cavernosal injection to improve erectile dysfunction However, in clinical treatment, there are still a considerable number of patients who do not respond well to these medications [23], and the lack of both can improve diabetes and can improve ED drugs. At present, the specific mechanism of erectile dysfunction caused by type 2 diabetes mellitus remains unclear, which is also the reason the poor effect of simply drugs to control blood glucose and treating erectile dysfunction with drugs is not satisfactory in clinical practice.Therefore, it is urgent to explore the mechanism of T2DMED, explore new drugs and new targets for improving T2DMED, and provide a new treatment idea for T2DMED patients.We found that the body weight, blood glucose, insulin resistance and erectile function of the treated mice were significantly decreased, and the expression of contractile phenotypic marker α-SMA was increased, while the synthetic phenotypic marker OPN was decreased, the corpus cavernosum smooth muscle fibers were increased, and PI3K/AKT/mTOR signaling pathway was activated.We established an in vitro model of spongy smooth muscle cultured with high oleic acid and high glucose and treated it with Nesfatin 1. We found that the CCSMCs phenotype changed to contractile after treatment, and the PI3K/AKT/mTOR signaling pathway was activated. After we added Esculetin to inhibit PI3K/AKT signaling pathway, the expression of α-SMA was significantly decreased and the expression of OPN was increased in corpus cavernosum smooth muscle cells. The effect of Nesfatin-1 on improving the phenotype switching of CCSMCs was significantly inhibited, which further demonstrated that Nesfatin-1 regulated the phenotype switching of CCSMCS through PI3K/AKT/mTOR signaling pathway.

Some studies have reported the use of the streptozotocin (STZ) injection method to induce diabetic erectile dysfunction in animal models, where the destruction of pancreatic islet cells leads to diabetes [[24], [25], [26]]. However, this approach does not align with the clinical epidemiological characteristics, given that type 2 diabetes mellitus is more prevalent in practical medical settings.In our research, we adopted a well-recognized modeling technique by continuously feeding mice with a high-fat diet for 16 weeks to establish a mouse model that closely mimics the disease characteristics and metabolic status of type 2 diabetes mellitus. Evaluation of relevant indicators of glucose metabolism in the model group revealed a significant increase in body weight, body type, and blood glucose values compared to the control group, showing statistically significant differences (P < 0.05).Subsequently, we measured the ICP in the mice. The ICP of the mice in the model group decreased, manifesting the typical characteristics of the T2DMED model. This outcome indicated that mice with type 2 diabetes mellitus induced solely by a high-fat diet would exhibit an erectile dysfunction phenotype, affirming the successful establishment of the T2DMED model.

We delved deeper into understanding the mechanism behind T2DMED. The phenotypes of CCSMCs can be categorized into contractile and synthetic types. Contractile CCSMCs exhibit considerable plasticity, allowing them to undergo phenotypic regulation from a contractile state to a synthetic state in response to local environmental stimul [27]. The characteristic of smooth muscle cell transition to the synthetic phenotype is an increase in expressions of extracellular matrix and type I collagen, while the expressions of contractile cytoskeletal proteins (such as α-SMA, SM22α, etc.) decrease and the expressions of synthetic cytoskeletal proteins (such as OPN, tenascin-C, etc) [28]. The main component of contractile smooth muscle cells is myofilament, which is an important factor in maintaining smooth muscle contraction function. The synthetic phenotype has a strong ability to synthesize extracellular matrix, manifesting as excessive proliferation and fibrosis of smooth muscle cells, which can cause contractile dysfunction.Our study found that type 2 diabetes mellitus leads to a significant reduction of α-SMA and a significant increase of OPN in corpus cavernosum smooth muscle cells, which is consistent with the transformation of corpus cavernosum smooth muscle cells into synthetic cells, which may be the mechanism of type 2 diabetes mellitus leading to ED.

In recent years, the significant role of Nesfatin-1 in regulating blood glucose and improving diabetes-related complications has been widely reported [29]. Previous studies have found that Nesfatin-1 can improve obesity-related type 2 diabetes and ameliorate atherosclerosis in the cardiovascular system [30].

Therefore, we hypothesize that Nesfatin-1 may not only improve type 2 diabetes but also alleviate T2DMED. Thus, we attempted to investigate the role of Nesfatin-1 in improving T2DMED and its specific mechanism using the T2DMED mouse model. Through exogenous administration of Nesfatin-1 to the model mice, we observed significant reductions in blood sugar, body weight, and body size, as well as significant improvements in insulin resistance and glucose tolerance. This means that Nesfatin-1 effectively corrected the metabolic disorder in the mice. Even more surprisingly, we found a significant improvement in erectile dysfunction in mice treated with Nesfatin-1, confirming the potential of Nesfatin-1 as a therapeutic option for T2DMED.

In further mechanistic exploration, our investigation revealed that Nesfatin-1 treatment in T2DMED mice resulted in an increase in the number of smooth muscle fibers, a significant upregulation in the expression of the contractile phenotype marker, and a substantial improvement in the contractility of the penile cavernosum. These findings suggest that Nesfatin-1 has the capacity to reduce α-SMA and increase OPN, inducing a transformation in the synthetic phenotype of corpus cavernosum smooth muscle towards the contractile phenotype. Consequently, this restoration of the normal contractile function of corpus cavernosum smooth muscle is indicative of the therapeutic potential of Nesfatin-1 in T2DMED.

To validate our findings, we conducted in vitro cell experiments. We cultured cavernous smooth muscle cells in a high-glucose and high-oleic acid environment to simulate the microenvironment of diabetic metabolic disorder. Our observations revealed that corpus cavernosum smooth muscle cells exhibited a synthetic phenotype consistent with those from type 2 diabetic mice when exposed to a culture environment with high oleic acid and high glucose. After Nesfatin-1 intervention, there was a transformation from a synthetic to a contractile type.

Upon further exploration of the mechanism, we observed that the PI3K/AKT/mTOR signaling pathway was significantly inhibited. In spongy smooth muscle cells cultured with high oleic acid and high glucose,α-SMA expression was significantly decreased, and OPN expression was significantly increased. Following Nesfatin-1 treatment, *p*-AKT and *p*-mTOR significantly increased, activating the PI3K/AKT/mTOR signaling pathway. Simultaneously, α-SMA expression significantly increased, and OPN expression significantly decreased.

To further confirm the role of the signaling pathway, we introduced Esculetin to Nesfatin-1 treated cells to directly inhibit the PI3K/AKT/mTOR signaling pathway. We found that Nesfatin-1 significantly inhibited the improvement of phenotype switching in cavernous smooth muscle cells. This further substantiates that Nesfatin-1 regulates the phenotypic switching of CCSMCs through the PI3K/AKT/mTOR signaling pathway.

In recent years, there has been a growing body of research on the role of the star protein Nesfatin-1. Most of these studies have focused on the significant impact of Nesfatin-1 on improving diabetes and obesity [[30], [31], [32]]. Currently, there is a lack of research investigating the role of Nesfatin-1 in the treatment of T2DMED. Erectile dysfunction, as a complication of type 2 diabetes, is associated with type 2 diabetes but also has independent pathological mechanisms. Whether Nesfatin-1 can ameliorate T2DMED and the mechanisms involved present crucial scientific questions.This study employed a high-fat feeding approach to establish a type 2 diabetes mouse model, confirming the occurrence of T2DMED. Leveraging cell experiments to simulate the glucose and lipid metabolism microenvironment of corpus cavernosum smooth muscle cells in type 2 diabetes, Nesfatin-1 was introduced for intervention and investigation. The findings demonstrate that Nesfatin-1 not only improves T2DMED but also plays a crucial role in the phenotypic transformation of CCSMCs.

Our study is the first to investigate the novel peptide, Nesfatin-1, which not only improves the metabolic microenvironment but also significantly regulates the phenotypic conversion of CCSMCs in the treatment of T2DMED. Furthermore, our study is the first to delve into the PI3K/AKT/mTOR metabolic pathway, which regulates the phenotypic conversion of cavernous smooth muscle cells, elucidating the positive role of the PI3K/AKT/mTOR signaling pathway in improving T2DMED.

However, there are also limitations to our study. The erection of the penis is not only related to the cavernous smooth muscle cells but also to the endothelial cells of the cavernous sinus, which play an important role in the erection. Our study only describes the important role of Nesfatin-1 in regulating the phenotypic conversion of cavernous smooth muscle cells in the treatment of T2DMED, without further studying whether Nesfatin-1 has a similar effect on improving T2DMED in endothelial cells of the cavernous sinus. Additionally, due to technical limitations, we were unable to directly measure the blood pressure in the hearts of live mice and ensure that they lived for a sufficient period of time. Instead, we measured the tail artery pressure indirectly to reflect blood pressure. To some extent, there is a slight difference between the actual blood pressure of the entire mouse body and the measured value, although this difference does not affect the accuracy of the experiment.

In conclusion, our study found and confirmed that Nesfatin-1 could improve both glucose metabolism disorders and diabetic Ed in T2DMED mice, and this effect may be mediated by Nesfatin-1 promoting the transformation of corpus cavernosus smooth muscle cells to contractile phenotype through the PI3K/AKT/mTOR signaling pathway. This study provides a new idea for the treatment of T2DMED.

## Statement

The study is reported in accordance with ARRIVE guidelines. All methods of this study were carried out in accordance with relevant guidelines and regulations, and all regulations were met.This study was approved by the Ethics Committee of Ningxia Medical University (2021-G067).

## Data availability statement

All data generated or analyzed during this study are included in this published article and its supplementary information files.

## CRediT authorship contribution statement

**Keming Chen:** Writing – review & editing, Writing – original draft, Validation, Software, Resources, Methodology, Funding acquisition, Formal analysis, Data curation, Conceptualization. **Bincheng Huang:** Conceptualization. **Jiajing Feng:** Conceptualization. **Shuzhe Fan:** Data curation, Conceptualization. **Zhengxing Hu:** Conceptualization. **Shuai Ren:** Conceptualization. **Haifu Tian:** Conceptualization. **A.L.-QAISIMOHAMMED Abdulkarem:** Writing – review & editing. **Xuehao Wang:** Conceptualization. **Yunshang Tuo:** Conceptualization. **Xiaoxia Liang:** Conceptualization. **Haibo Xie:** Conceptualization. **Rui He:** Writing – review & editing, Writing – original draft, Data curation, Conceptualization. **Guangyong Li:** Writing – review & editing, Writing – original draft, Validation, Project administration, Methodology, Data curation, Conceptualization.

## Declaration of competing interest

The authors declare the following financial interests/personal relationships which may be considered as potential competing interests:Guangyong Li reports financial support, administrative support, article publishing charges, equipment, drugs, or supplies, statistical analysis, and writing assistance were provided by 10.13039/501100001809National Natural Science Foundation of China. Guangyong Li reports financial support was provided by Ningxia Key 10.13039/100006190Research and Development Project. Guangyong Li reports financial support was provided by Ningxia Natural ScienceFoundation. Guangyong Li reports financial support was provided by Ningxia science and technology innovation leading talent training project. Guangyong Li reports was provided by Ningxia Medical University research project. Guangyong Li reports a relationship with 10.13039/501100001809National Natural Science Foundation of China that includes: funding grants. Guangyong Li reports a relationship with 10.13039/100016692Key Research and Development Program of Ningxia that includes: funding grants. Guangyong Li reports a relationship with Ningxia Hui Autonomous Region Natural ScienceFoundation that includes: funding grants. Guangyong Li reports a relationship with Ningxia science and technology innovation leading talent training project that includes: funding grants. Guangyong Li reports a relationship with 10.13039/501100004179Ningxia Medical University research project that includes: funding grants. If there are other authors, they declare that they have no known competing financial interests or personal relationships that could have appeared to influence the work reported in this paper.
