# Supplementary figures and images for: Nesfatin-1 regulates the phenotype transition of cavernous smooth muscle cells by activating PI3K/AKT/mTOR signaling pathway to improve diabetic erectile dysfunction
Source: Heliyon. 2024 Jun 16;10(13):e32524. doi: 10.1016/j.heliyon.2024.e32524 (PMC467047; doi:10.1016/j.heliyon.2024.e32524)

Fig 4(c)

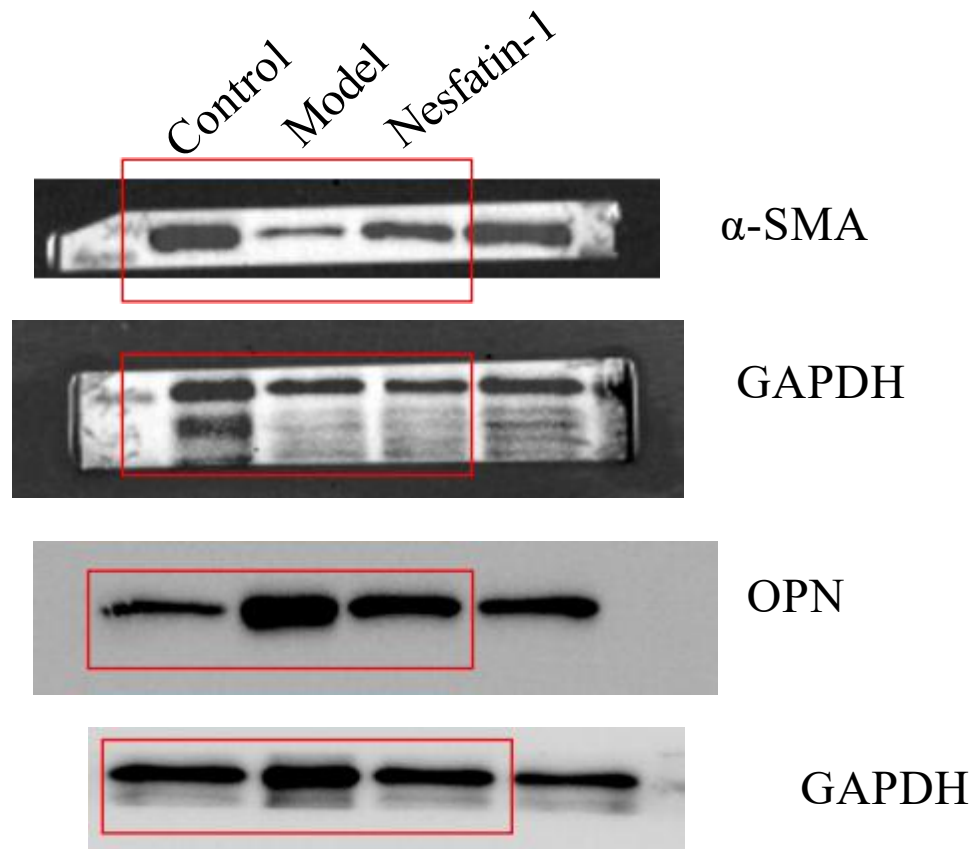

Fig5(a)

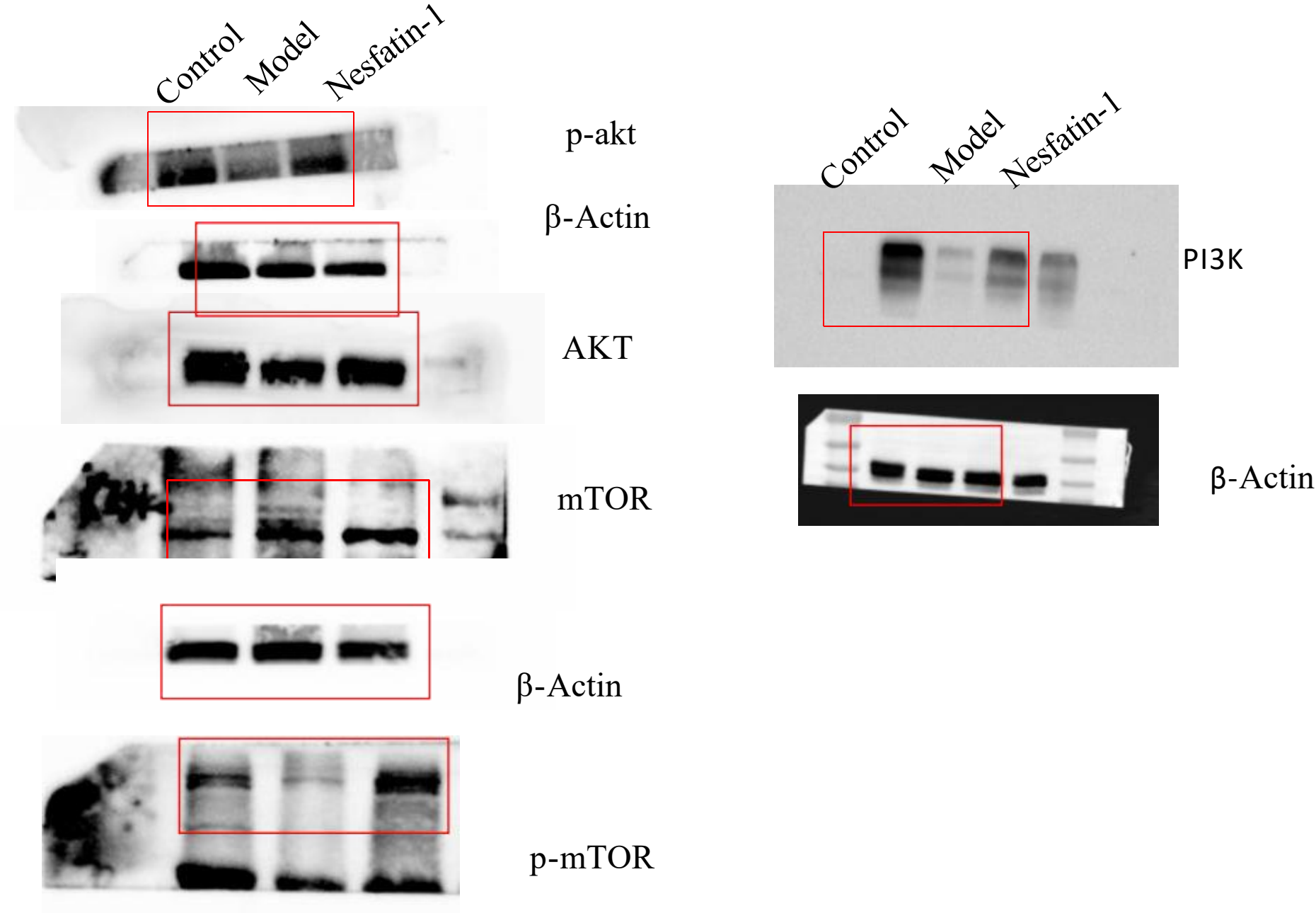

Fig7(c)

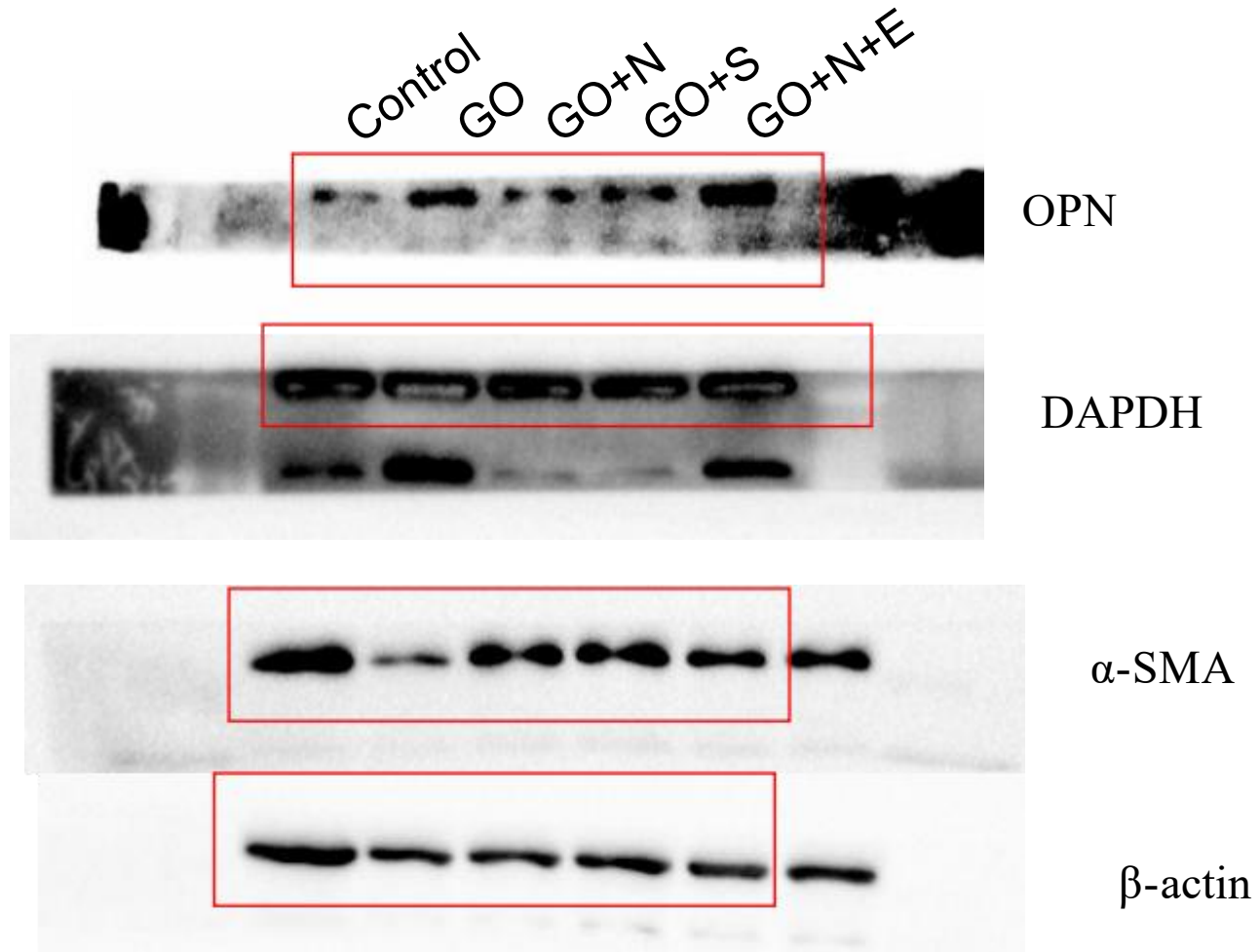

Fig7(e)

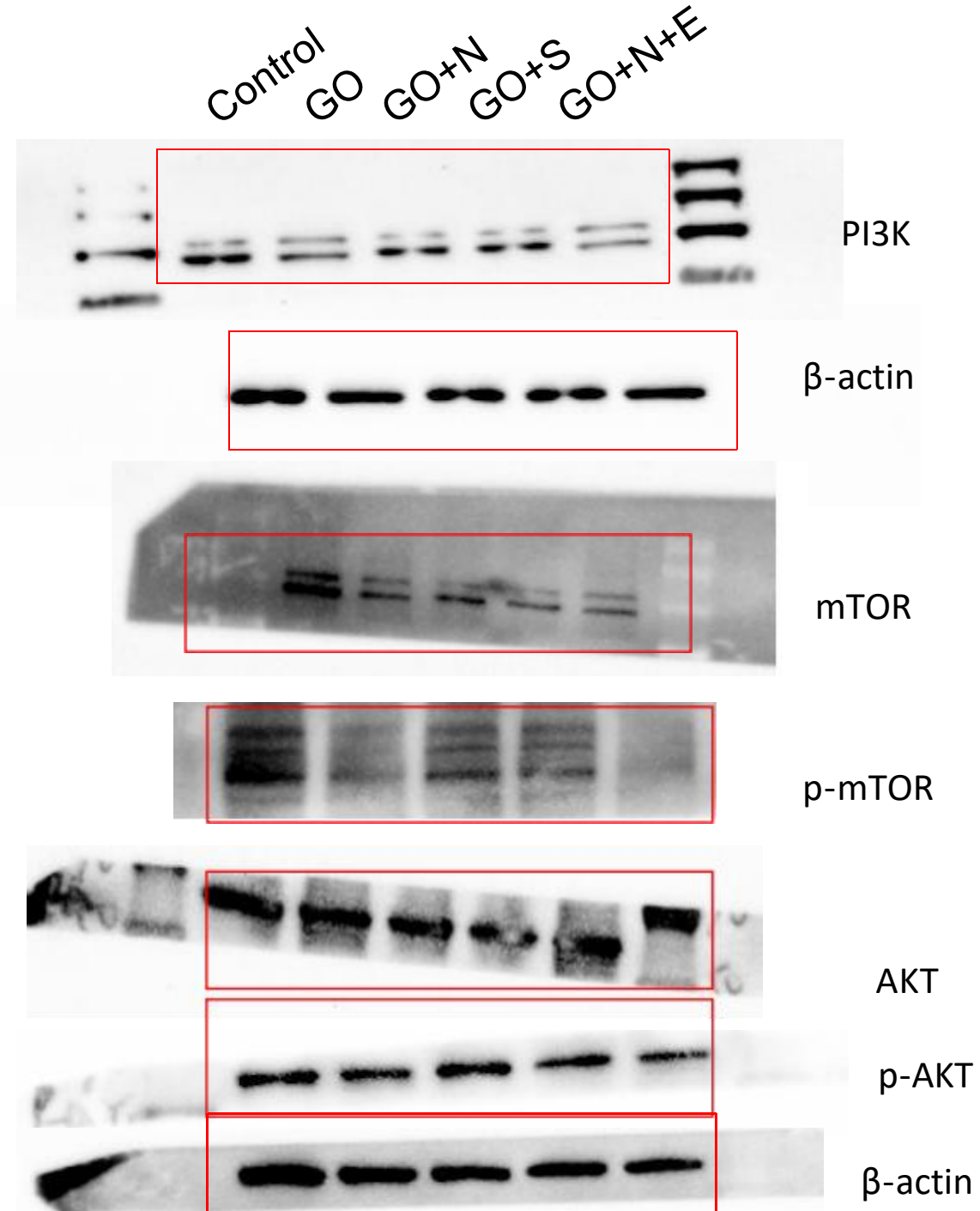

Supplement: Multimedia component 1 [file mmc1.pdf]
