# Supplementary material for: Nesfatin-1 regulates the phenotype transition of cavernous smooth muscle cells by activating PI3K/AKT/mTOR signaling pathway to improve diabetic erectile dysfunction
Source: Heliyon. 2024 Jun 16;10(13):e32524. doi: 10.1016/j.heliyon.2024.e32524 (PMC467047; doi:10.1016/j.heliyon.2024.e32524)
Supplement: Multimedia component 2 [file mmc2.pdf]

# Mass

| week | Control | Control | Control | Control | Control | Model | Model | Model | Model | Model |
|---|---|---|---|---|---|---|---|---|---|---|
| 0 | 19.3 | 18.9 | 18.3 | 18.1 | 19.1 | 22.5 | 21.4 | 20.4 | 20.6 | 20.1 |
| 1 | 19.6 | 18.5 | 19 | 18.2 | 20.3 | 27.1 | 26.4 | 24.2 | 23.3 | 24.9 |
| 2 | 22.5 | 21.7 | 22 | 21.9 | 22.8 | 28.7 | 28.4 | 26.5 | 24.5 | 26.5 |
| 3 | 23.4 | 22.9 | 23 | 23.7 | 23.7 | 31.8 | 32 | 29.6 | 27 | 29.4 |
| 4 | 23.5 | 23.8 | 24.2 | 24.8 | 24.5 | 34.3 | 34.2 | 32 | 28.9 | 31.6 |
| 5 | 24.2 | 23.3 | 24.7 | 24.6 | 25.2 | 36.6 | 37.1 | 35.4 | 31.4 | 34.4 |
| 6 | 25.7 | 25 | 25.5 | 25.9 | 25.4 | 39.2 | 40.4 | 38.9 | 34 | 37.8 |
| 7 | 27 | 25.7 | 25.7 | 26.8 | 25.8 | 42.5 | 43 | 41.1 | 35.8 | 39.8 |
| 8 | 28 | 26.6 | 27 | 27.9 | 27.1 | 45 | 46.5 | 44.7 | 38.2 | 42.5 |
| 9 | 29.8 | 27.4 | 28.1 | 28.7 | 27.1 | 47.7 | 48.1 | 48.3 | 40.6 | 45.3 |
| 10 | 30.3 | 28.1 | 28.9 | 29.4 | 28.7 | 50.6 | 49 | 49.7 | 43.6 | 47.1 |
| 11 | 30.9 | 28.6 | 29.1 | 29.9 | 28 | 51.4 | 50.4 | 51.3 | 46 | 48.7 |
| 12 | 31.5 | 28.9 | 29.5 | 30.5 | 30.6 | 52.6 | 50.8 | 51.7 | 48.5 | 50.1 |
| 13 | 31.4 | 29.1 | 29.2 | 30.6 | 28.9 | 54.1 | 51.5 | 52 | 50.9 | 50.2 |
| 14 | 31.2 | 28.5 | 28.8 | 30.3 | 28.1 | 54 | 52.1 | 52.9 | 53.2 | 51.2 |
| 15 | 31.3 | 29.6 | 29.7 | 31.6 | 29 | 52.8 | 53 | 53.1 | 53.4 | 51.4 |
| 16 | 31.6 | 29.5 | 31 | 31.8 | 29.3 | 53.6 | 53.1 | 52.5 | 54.1 | 51.1 |

ICP/MSBP

| | Control | Control | Control | Model | Model | Model | Nesfatin-1 | Nesfatin-1 | Nesfatin-1 |
|---|---|---|---|---|---|---|---|---|---|
| ICP/MSBP | 0.959770115 | 0.934640523 | 0.939393939 | 0.484848485 | 0.574712644 | 0.492537313 | 0.952380952 | 0.833333333 | 0.982248521 |

ITT

| Time（mim） | Control | Control | Control | Control | Model | Model | Model | Model | Nesfatin-1 | Nesfatin-1 | Nesfatin-1 | Nesfatin-1 |
|---|---|---|---|---|---|---|---|---|---|---|---|---|
| 0 | 9.6 | 7.4 | 8.9 | 6.2 | 8 | 10.9 | 10.9 | 7.3 | 8.6 | 8.4 | 9.6 | 9.2 |
| 15 | 5.1 | 5.4 | 6.2 | 3.9 | 8.2 | 8.2 | 8.2 | 6.4 | 8.6 | 5.7 | 6 | 7 |
| 30 | 4.7 | 3.7 | 4.1 | 3.8 | 8.1 | 7.9 | 7.7 | 7.2 | 5.8 | 4.6 | 3.4 | 5.7 |
| 60 | 3.5 | 3.4 | 3.2 | 3.3 | 9.8 | 7.7 | 8.7 | 9.8 | 5 | 4.9 | 3.8 | 3.9 |
| 120 | 6 | 3.6 | 4.2 | 5.3 | 9.9 | 13.2 | 10.6 | 10 | 5.8 | 7.8 | 5.4 | 4.1 |

OGTT

| Time（mim） | Control | Control | Control | Control | Model | Model | Model | Model | Nesfatin-1 | Nesfatin-1 | Nesfatin-1 | Nesfatin-1 |
|---|---|---|---|---|---|---|---|---|---|---|---|---|
| 0 | 6.7 | 5.4 | 6.5 | 6.7 | 7 | 7 | 7.7 | 9.9 | 12.2 | 11.6 | 5.8 | 5.5 |
| 15 | 14.7 | 12.5 | 15.4 | 14.1 | 17.2 | 20.9 | 17.1 | 19.9 | 22.6 | 23.6 | 12.5 | 14.3 |
| 30 | 18.4 | 12.3 | 13.9 | 13.4 | 19.8 | 22.4 | 17.4 | 18.9 | 14 | 18.3 | 14 | 18.7 |
| 60 | 17.5 | 14.1 | 13.7 | 13.1 | 20 | 20.5 | 18.3 | 17.7 | 13 | 13.2 | 18 | 15.1 |
| 120 | 8.5 | 8.5 | 11.7 | 9 | 18.3 | 12.9 | 14.1 | 19.8 | 11.8 | 11.5 | 12.2 | 10 |

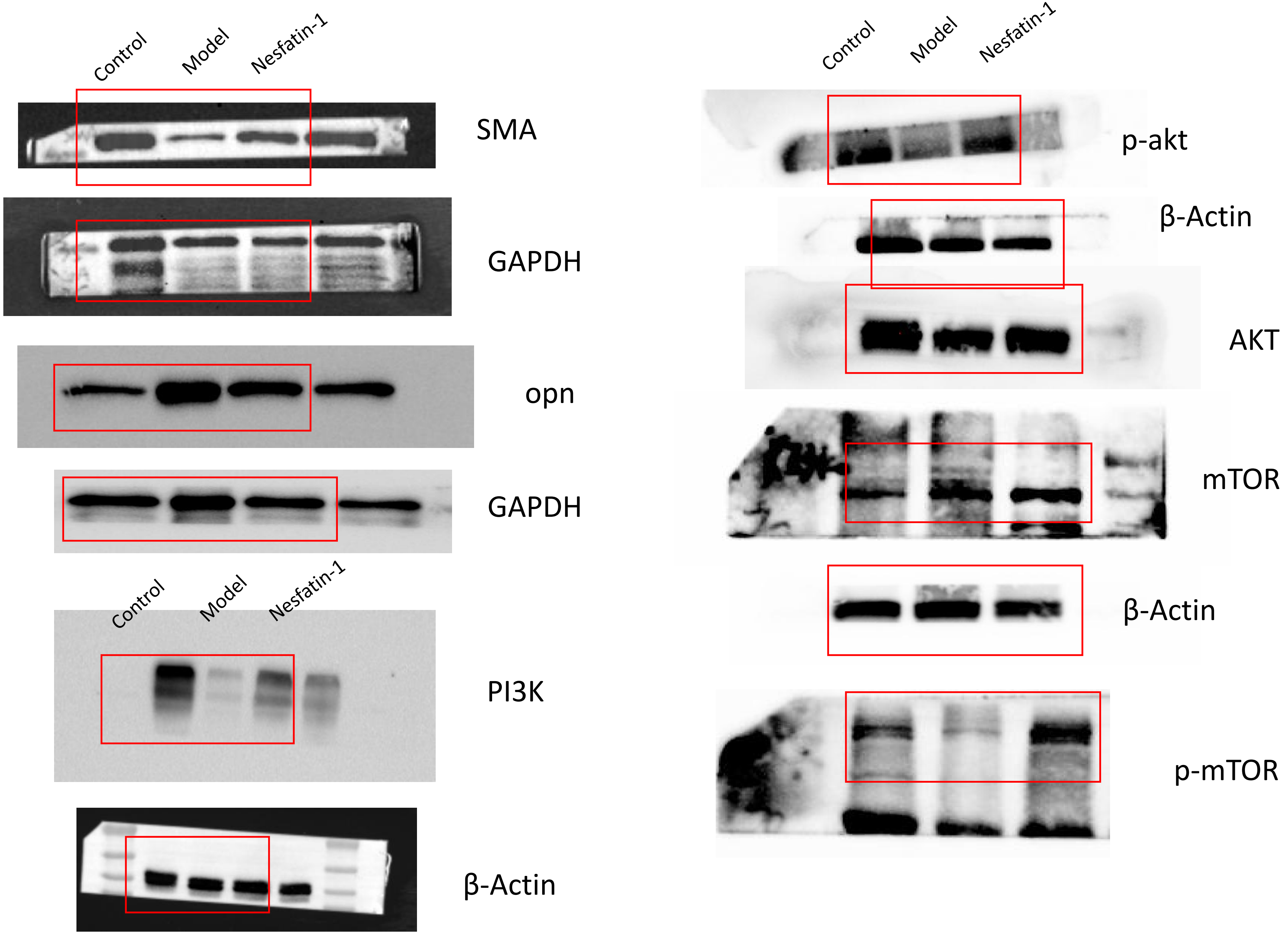

Control
Model
Nesfatin-1
SMA
GAPDH
opn
GAPDH
Control
Model
Nesfatin-1
PI3K
β-Actin
Control
Model
Nesfatin-1
p-akt
β-Actin
AKT
mTOR
β-Actin
p-mTOR

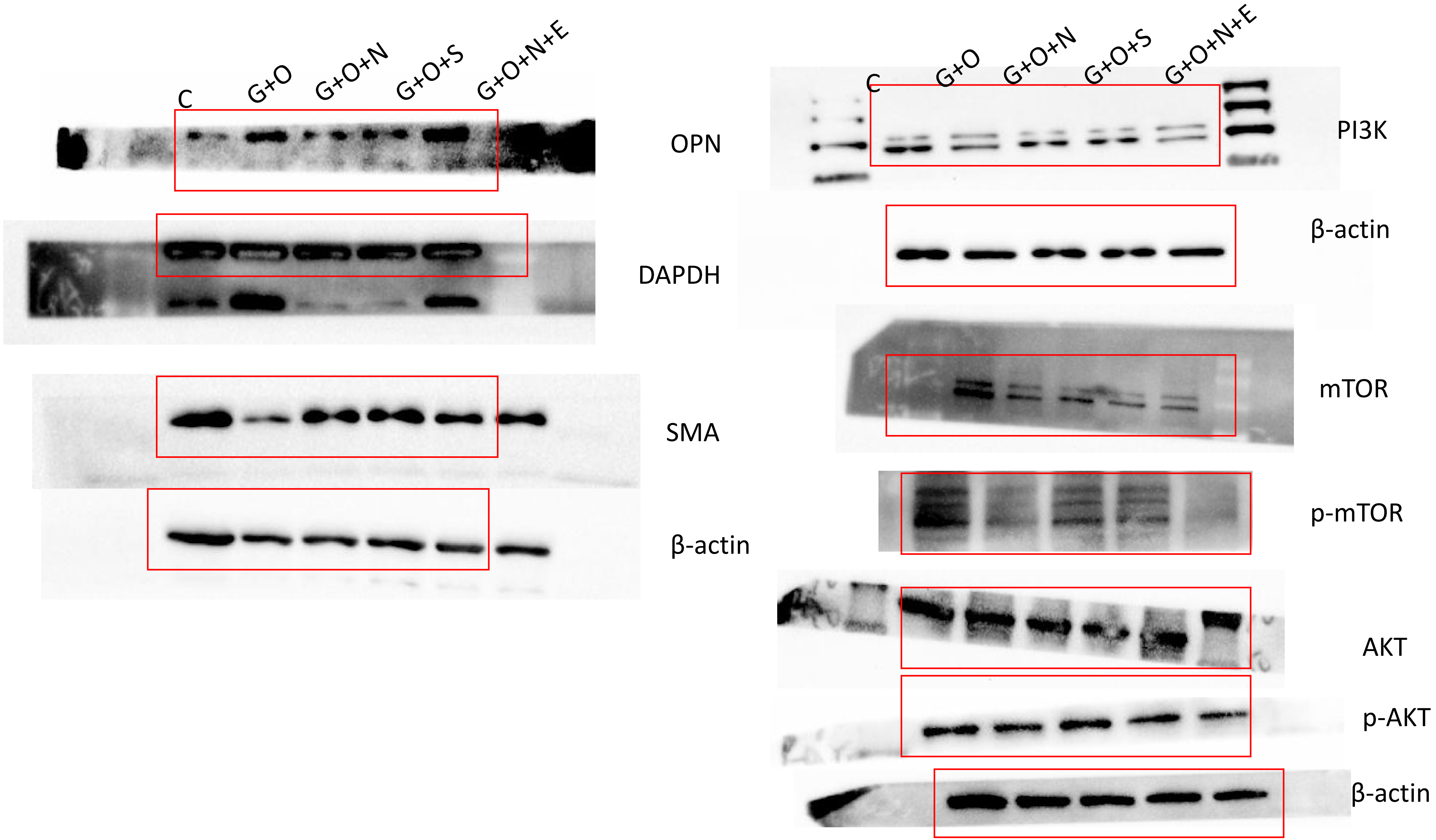
C
G+O
G+O+N
G+O+S
G+O+N+E
OPN
DAPDH
SMA
β-actin
PI3K
β-actin
mTOR
p-mTOR
AKT
p-AKT
β-actin

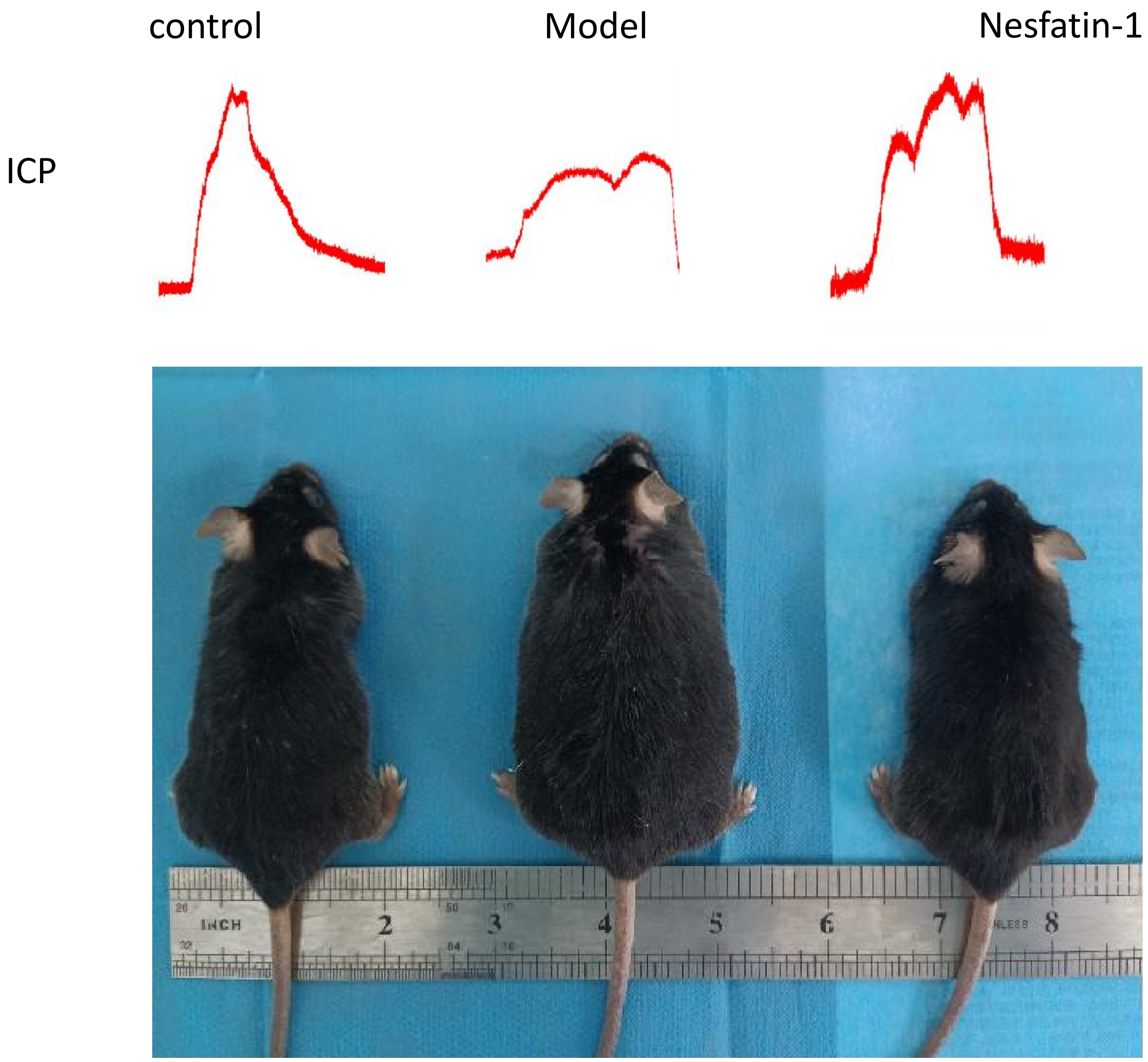
control
Model
Nesfatin-1
ICP
INCH
2
3
4
5
6
7
8

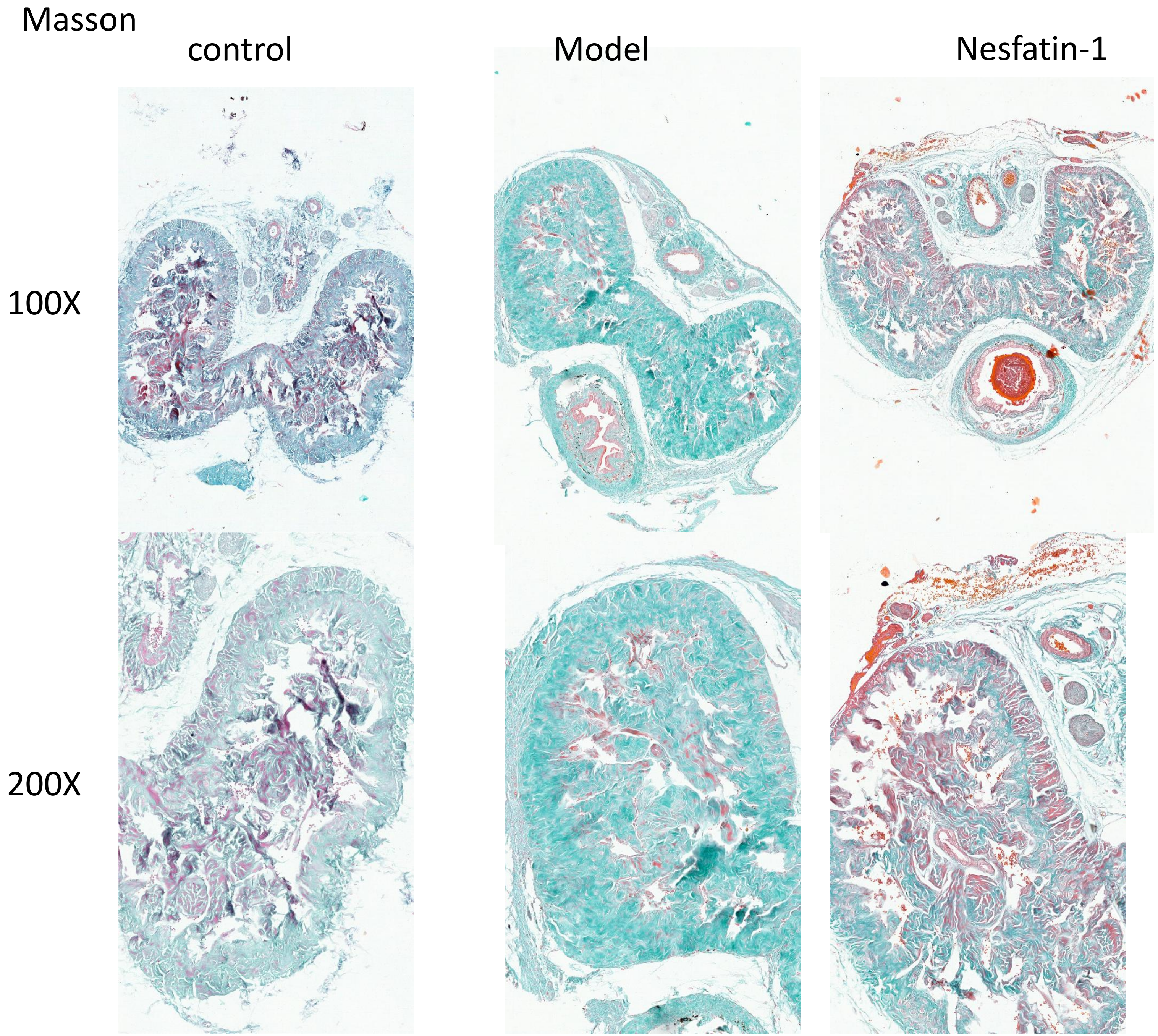
Masson
control
Model
Nesfatin-1
100X
200X

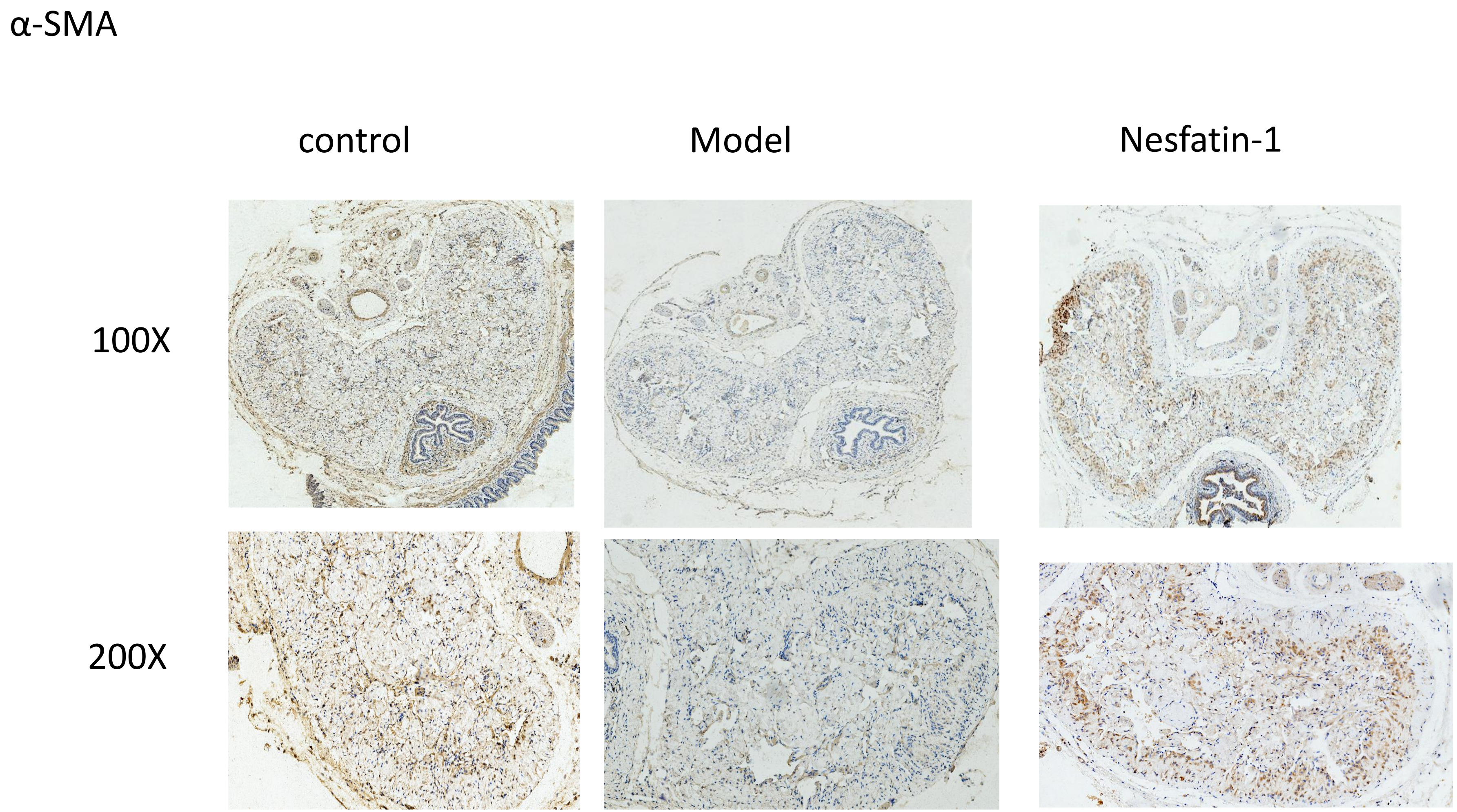
α-SMA
control
Model
Nesfatin-1
100X
200X

OPN

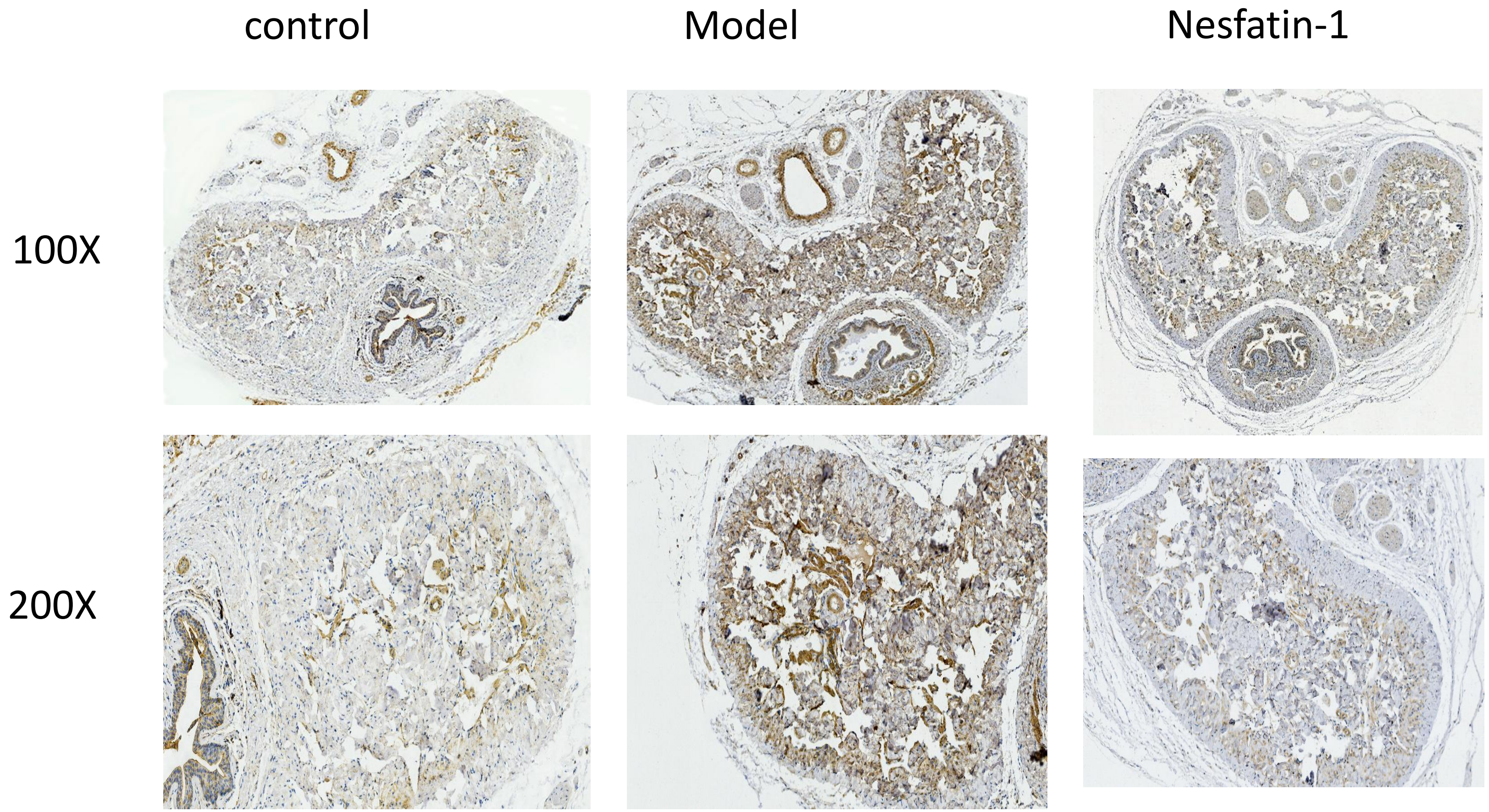

ICP

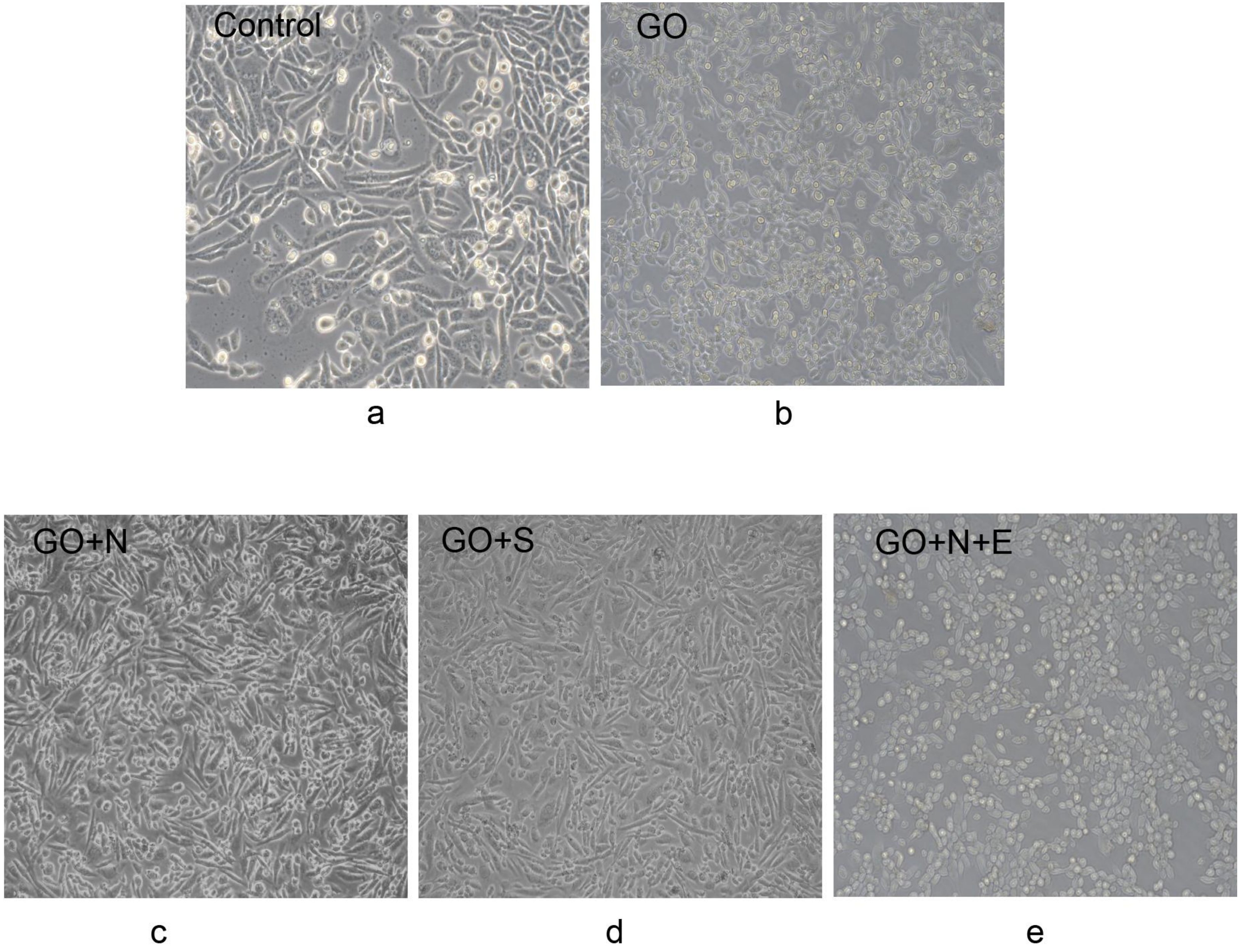

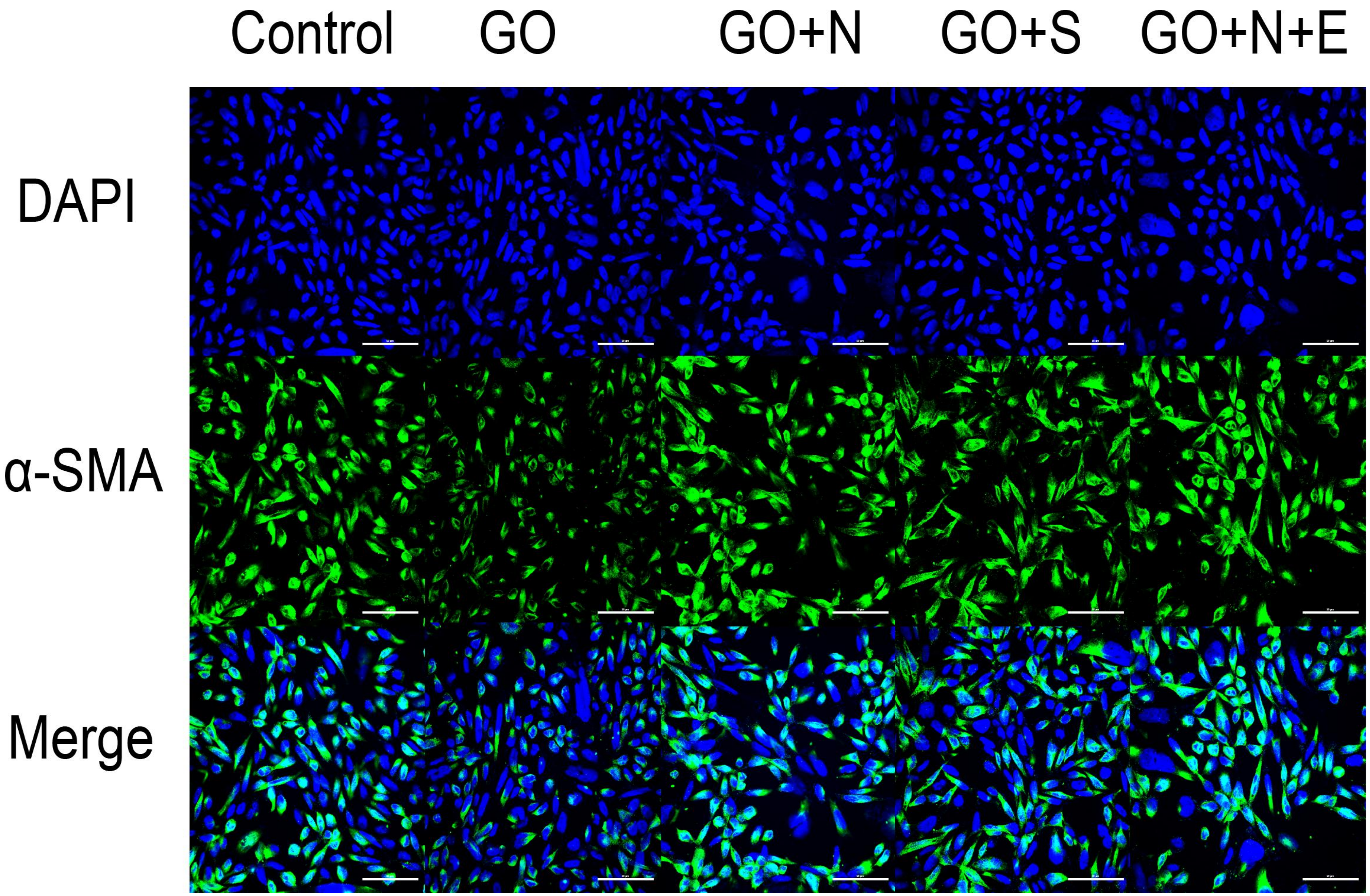
Control
GO
GO+N
GO+S
GO+N+E
DAPI
α-SMA
Merge

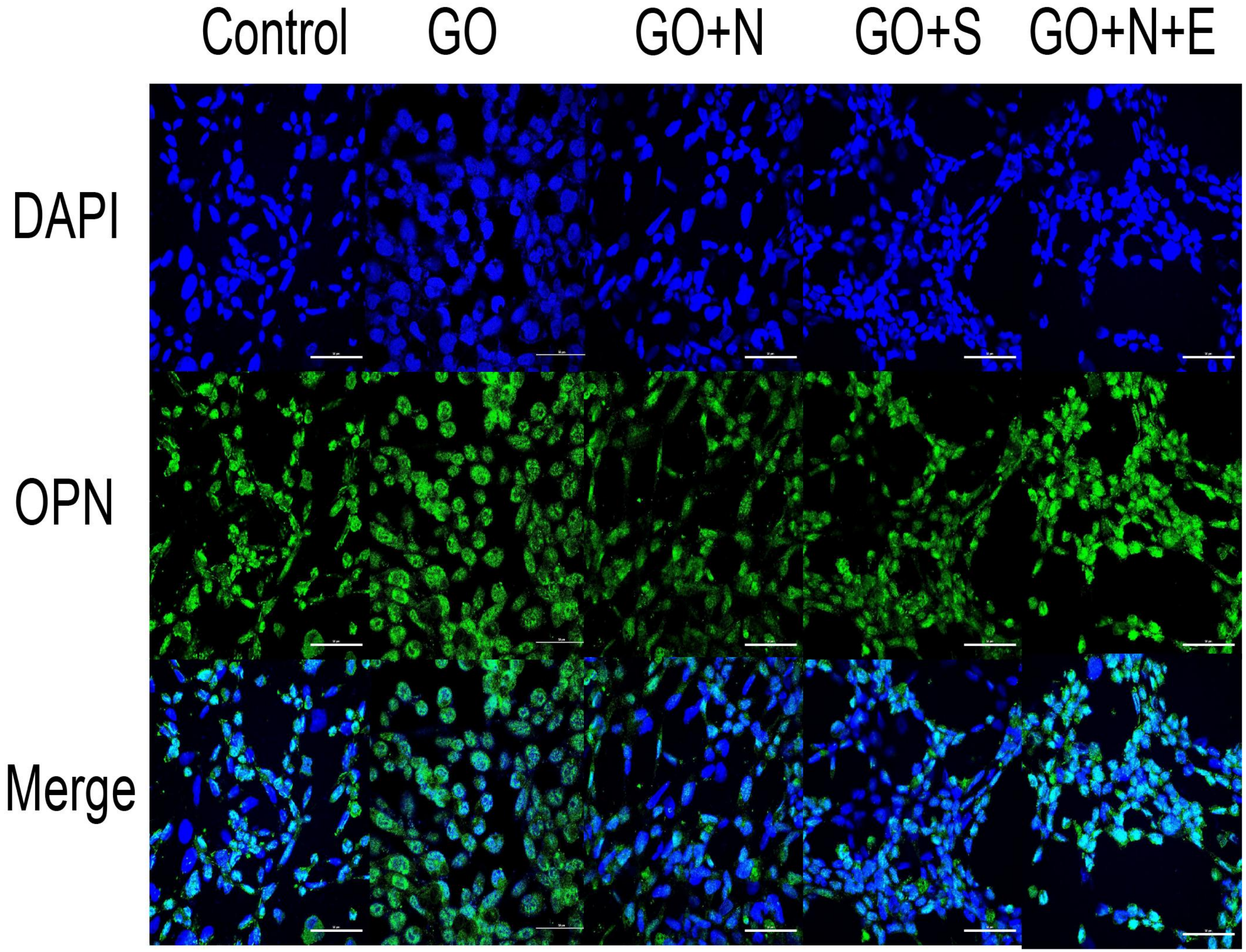
Control
GO
GO+N
GO+S
GO+N+E
DAPI
OPN
Merge
